# Prenatal screening in the era of non-invasive prenatal testing: a Nationwide cross-sectional survey of obstetrician knowledge, attitudes and clinical practice

**DOI:** 10.1186/s12884-020-03279-y

**Published:** 2020-10-01

**Authors:** Liying Yang, Wei Ching Tan

**Affiliations:** Department of Obstetrics & Gynaecology, Singapore General Hospital, 1 Hospital Drive, Singapore, 169608 Singapore

**Keywords:** Prenatal screening, First trimester screening, Non-invasive prenatal testing, Non-invasive prenatal screening, Trisomy 21, Down syndrome

## Abstract

**Background:**

Non-invasive prenatal testing (NIPT) has revolutionized the prenatal screening landscape with its high accuracy and low false positive rate for detecting Trisomy 21, 18 and 13. Good understanding of its benefits and limitations is crucial for obstetricians to provide effective counselling and make informed decisions about its use. This study aimed to evaluate obstetrician knowledge and attitudes regarding NIPT for screening for the common trisomies, explore how obstetricians integrated NIPT into first-line and contingent screening, and determine whether expanded use of NIPT to screen for sex chromosome aneuploidies (SCAs) and microdeletion/microduplication syndromes (CNVs) was widespread.

**Methods:**

A questionnaire was designed and administered with reference to the CHERRIES criteria for online surveys. Doctors on the Obstetrics & Gynaecology trainee and specialist registers were invited to participate. Medians and 95% confidence intervals (CI) were reported for confidence and knowledge scores.

**Results:**

94/306 (30.7%) doctors responded to the survey. First trimester screening (FTS) remained the main method offered to screen for the common trisomies. 45.7% (43/94) offered NIPT as an alternative first-line screen for singletons and 30.9% (29/94) for monochorionic diamniotic twins. A significant proportion offered concurrent NT and NIPT (25/94, 26.6%), or FTS and NIPT (33/94, 35.1%) in singletons. Varying follow up strategies were offered at intermediate, high and very-high FTS risk cut-offs for Trisomy 21. Respondents were likely to offer screening for SCAs and CNVs to give patients autonomy of choice (53/94, 56.4% SCAs, 47/94, 50% CNVs) at no additional cost (52/94, 55.3% SCAs, 39/94, 41.5% CNVs). Median clinical knowledge scores were high (10/12) and did not differ significantly between specialists (95% CI 10–11) and non-specialists (95% CI 9.89–11). Lower scores were observed for scenarios in which NIPT would be more likely to fail.

**Conclusions:**

Our findings show the diversity of clinical practice with regard to the incorporation of NIPT into prenatal screening algorithms, and suggest that the use of NIPT both as a first-line screening tool in the general obstetric population, and to screen for SCAs and CNVs, is becoming increasingly prevalent. Clear guidance and continuing educational support are essential for providers in this rapidly evolving field.

## Background

Non-invasive prenatal testing (NIPT) with cell-free DNA (cfDNA) has revolutionized the prenatal screening landscape for Trisomy 21, 18 and 13 with its high accuracy and low false positive rate for detecting these conditions [1]. The positive predictive values (PPVs) for these common trisomies are also superior to first trimester screening (FTS) for both low and high-risk women, and the negative predictive values (NPVs) approach 100% [2–4].

These performance characteristics, along with the ease of administration and non-invasive nature of NIPT, have increased its popularity for prenatal screening. There are multiple NIPT laboratories with varying clinical and reporting standards advertising their services to both providers and patients [5]. It is crucial that obstetricians offering NIPT have a good understanding of how the test works and its benefits and limitations, so that accurate counselling and interpretation of results can be provided. For instance, a foetal fraction of at least 4% is essential for a reportable result. A low foetal fraction is more likely in certain populations such as obese women and those with certain aneuploidies [6–8], and these facts need to be incorporated into pre and post-test counselling.

There is currently no consensus on the best way in which to incorporate NIPT into prenatal screening algorithms [9]. Some obstetricians offer NIPT alone, others in conjunction with nuchal translucency measurement (NT) or FTS, and others as a contingent test at varying FTS cut-offs. Each of these approaches have their respective advantages and trade-offs which obstetricians need to be cognizant of. Performing NIPT alone, for example, has potential drawbacks such as the risks of missing severe structural abnormalities that can be detected on first trimester ultrasound and atypical chromosomal abnormalities (e.g. triploidy) that could have been detected via FTS [10, 11].

Rapid technological advances have widened the scope of NIPT to include screening for sex chromosomal aneuploidies (SCAs) and microdeletion and microduplication syndromes (CNVs). Expanded screening is controversial, with proponents arguing that it allows for improved detection and early intervention for affected children, and opponents countering that the tests are insufficiently validated to allow for reliable genetic counselling and increase the overall false positive rate [12, 13]. A good understanding of these issues is crucial for obstetricians to appropriately advise their patients.

This study was conceived to evaluate current obstetrician knowledge and attitudes regarding NIPT for screening for the common trisomies. We also aimed to explore how obstetricians incorporated NIPT into prenatal screening algorithms for Trisomy 21, 18 and 13, and whether use of NIPT to screen for SCAs and CNVs was widespread. This information was felt to be valuable for understanding current practice and identifying areas that would benefit from improved educational efforts and/or updated clinical guidance.

## Methods

### Study setting

All pregnant women are offered the option of prenatal screening for Down syndrome in Singapore [14]. They are free to choose from the range of tests available which include nuchal translucency, first trimester screening, NIPT or a combination of these. The tests are self-funded and the price varies depending on the setting in which they are seen, whether in public or private hospitals. The cost for FTS with government subsidy is around USD$180 and NIPT approximately USD$525. Protocols for pre and post-test counselling are centre-based and not uniform; information on Down syndrome screening and explanation of the results may be provided by either the ordering obstetrician or a genetic counsellor depending on the institution.

### Recruitment of participants

All Obstetrics & Gynaecology trainees and members of the College of Obstetrics & Gynaecology with an active obstetric practice were invited via email to participate in the study in April 2020. These two groups encompass the vast majority of practicing obstetricians from both the public and private sectors in Singapore. Reminder emails were sent two and four weeks after the initial invitation.

### Questionnaire development

A 3-part online questionnaire (Additional File 1) was designed based on literature review and questionnaires used in previous studies [9, 15–17]. It was reviewed for content validity by three consultants in maternal-foetal medicine (MFM) and edited for clarity after pilot-testing on a group of 15 obstetricians.

The questionnaire started with a cover page describing the aims of our study, consent and confidentiality. It was administered with reference to the CHERRIES criteria [18] for online surveys *(*Additional File 2*).* It collected demographic details and had two sections to explore the themes of attitudes and clinical practice, as well as clinical knowledge. Question formats included multiple choice, “true or false” statements and Likert scales.

### Data analysis

Data was stored and analysed using STATA/SE 15.0 for Windows. Statistical analysis for confidence and knowledge scores was done by calculating medians and 95% confidence intervals.

### Ethical considerations

This study was approved by the Singhealth Institutional Review Board. All responses were recorded anonymously and no identifying information was collected. Consent to participate in the study was implicit in the completion and submission of the questionnaire, as stated on the study cover page.

## Results

### Demographics

A total of 94 out of 306 obstetricians participated in our study, yielding a response rate of 30.7%. Their demographic characteristics are summarized in Table 1. 63/94 (67%) of respondents worked in the public sector and 55/94 (58.5%) were consultant obstetricians. 79/94 (84%) of the respondents reported 25–75% of their practice being in obstetrics.

**Table 1:** Demographic distribution of respondents

Question	Number (%)
Practice setting	
Public	63 (67)
Private	31 (33)
Level of expertise	
Medical officer	21 (22.3)
Registrar	18 (19.1)
Consultant	55 (58.5)
Years in practice	
4 or less	12 (12.8)
5–10	41 (43.6)
11–15	10 (10.6)
> 15	31 (33)
Percentage of practice in obstetrics	
< 25%	5 (5.3)
25–50%	36 (38.3)
50–75%	43 (45.7)
> 75%	10 (10.6)
Maternal-foetal medicine (MFM) specialist	
Yes	11 (11.7)
No	83 (88.3)

Non-specialists and consultants in the public sector had higher response rates than specialists and consultants in the private sector; the former made up 41.5% (39/94) of our respondents compared with 23.9% (73/306) of the invited cohort, while consultants in the public sector made up 25.5% (24/94) of our respondents compared with 18.6% (57/306) of the invited cohort.

### Confidence in providing pre and post-test Counselling

Obstetricians were asked how confident they felt in counselling patients for prenatal screening, including discussing the clinical features of Trisomy 21, the performance of NIPT and the options if patients receive high risk NIPT results. The responses were recorded using a 5-point Likert scale, with “1” being not comfortable and “5” being very comfortable. The majority of participants reported being comfortable discussing the clinical features of Trisomy 21 (median confidence score 5), the accuracy and limitations of NIPT for Trisomy 21, 18 and 13 and SCAs (median confidence score 4) and options if patients received high risk NIPT results (median confidence score 4). Median confidence scores and score distributions for each of the questions are tabulated in Table 2.

**Table 2:** Confidence in providing Pre and Post-Test Counselling

How comfortable are you with discussing the:	Median Confidence Scores (Range)
Clinical features of Trisomy 21	5 (3–5)
Accuracy and limitations of NIPT for T21, T18 and T13	4 (2–5)
Accuracy and limitations of NIPT for SCAs	4 (2–5)
Options if patient receives a high risk NIPT result	4 (1–5)

Median total confidence scores were higher among MFM specialists (19/20, 95% CI 18–19.29) versus non-MFM specialists (17/20, 95% CI 16–17), and specialists felt more confident providing pre and post-test counselling (18/20, 95% CI 17.7–19) than non-specialists (16/20, 95% CI 15–16.1).

### Clinical practice

Respondents were asked about their primary aim in offering prenatal screening for their general obstetric patients. The majority of respondents reported that their primary intention was to provide screening for the common foetal aneuploidies (67/94, 71.3%). Fewer participants reported that their main aim was to provide screening for Trisomy 21 alone (13/94, 13.8%) and screening for as many genetic conditions as possible (11/94, 11.7%).

Respondents were asked about their usual clinical practice in offering prenatal screening for Down syndrome to their general obstetric patients. Three scenarios of a singleton pregnancy, monochorionic diamniotic (MCDA) twins and dichorionic diamniotic (DCDA) twins were offered and participants were asked to select whether they would offer NT alone, FTS, NIPT, NT in combination with NIPT or FTS in combination with NIPT. Respondents were allowed to select more than one option for each scenario. The responses are summarized in Table 3.

**Table 3:** Options offered for Down syndrome screening in the first trimester

	Singleton Foetus N (%)	DCDA Twins N (%)	MCDA Twins N (%)
**Nuchal translucency (NT) alone**	7 (7.4)	16 (17)	13 (13.8)
**First trimester screening (FTS) comprising NT, PAPPA, bHCG)**	74 (78.7)	65 (69.1)	64 (68.1)
**NIPT**	43 (45.7)	16 (17)	29 (30.9)
**NT in combination with NIPT**	25 (26.6)	15 (16)	13 (13.8)
**FTS in combination with NIPT**	33 (35.1)	11 (11.7)	20 (21.3)
**Others**	Invasive testing: 2 (2.1) Two-step contingency testing 1 (1.7)	Invasive testing: 2 (2.1)	Invasive testing: 2 (2.1)

The most frequently utilized NIPT platform was Harmony, used by 68/94 (72.3%) of respondents. The other frequently used platforms were Panorama (26/94, 27.7%) and iGene (16/94, 17%). The main reasons given for selection of particular NIPT platforms were based on institution availability (69/94, 73.1%), availability of post-test support such as provision of counselling for no-call results (28/94, 29.8%), lower cost (15/94, 16%) and perceived superior test performance (12/94, 12.8%).

**Fig. 1:**
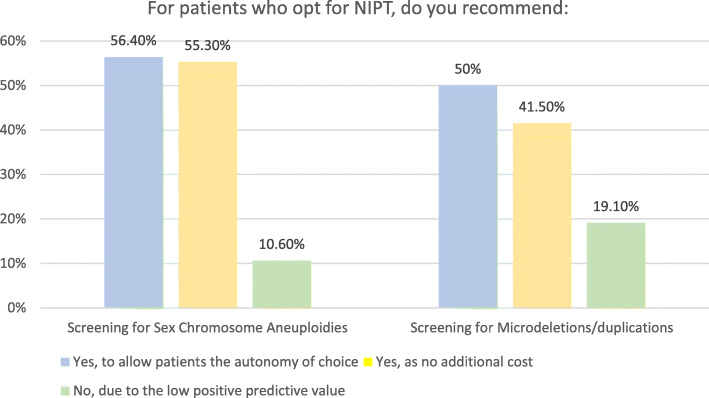
NIPT for sex chromosome aneuploidies and microdeletions/duplications

Most respondents offered their patients screening for SCAs and microdeletions and microduplications. Without comprehensive pre-test counselling, this practice can be potentially unsafe with ethical implications. The main reasons for offering screening for SCAs included wanting to give patients the autonomy of choice (53/94, 56.4%) and no additional cost (52/94, 55.3%). Similarly, most respondents offered screening for microdeletions and microduplications to give patients the autonomy of choice (47/94, 50%) at no additional cost (39/94, 41.5%). Less than one fifth of the respondents did not recommend using NIPT to screen for microdeletions and microduplications (18/94, 19.1%) and cited the low positive predictive value as the main reason. The results are summarized in Fig. 1.

### Recommendations at various FTS risk levels

Respondents were asked what they would offer patients with intermediate, high and very high-risk results on FTS. Respondents were instructed to select all applicable answers for each scenario. The responses are summarized in Table 4 below. Two respondents indicated that they would offer termination of pregnancy for a very high risk [1, 7] on FTS.

**Table 4:** Options offered at varying FTS risk levels

	No further testing	NIPT	Invasive testing	Termination of Pregnancy
The adjusted risk for T21 is 1:670 on FTS (Intermediate risk)	28 (29.8%)	89 (94.7%)	14 (14.9%)	0 (0%)
The adjusted risk for T21 is 1:86 on FTS (High risk)	11 (11.7%)	73 (77.7%)	81 (86.2%)	0 (0%)
The adjusted risk for T21 is 1:7 on FTS (Very high risk)	11 (11.7%)	20 (21.3%)	92 (97.9%)	2 (2.1%)

### Clinical knowledge

The median score for the clinical knowledge section was 10 (95% CI 10–11) out of a maximum total score of 12. There was no significant difference in median scores between specialists (95% CI 10–11) and non-specialists (95% CI 9.89–11), or between MFM (95% CI 9.7–12) and non-MFM doctors (95% CI 10–11). The overall scores and breakdown of the clinical knowledge scores by question are presented in Table 5 and Table 6.

**Table 5:** Overall scores

Clinical Knowledge Score	Median (95% CI)
Overall	10 (10–11)
MFM	11 (9.7–12)
Non-MFM	10 (10–11)
Specialists	11 (10–11)
Non-Specialists	10 (9.89–11)

**Table 6:** Clinical Knowledge Scores

	Correct Answer	Correct response, n (%)
Where does cell-free DNA originate from?	Placenta cells	74 (78.7%)
NIPT can be offered from 8 weeks of pregnancy.	False	86 (91.5%)
All the chromosomal abnormalities diagnosed via amniocentesis can be detected via NIPT.	False	91 (96.8%)
NIPT is a diagnostic test for Trisomy 21.	False	91 (96.8%)
A high risk NIPT result should be confirmed with invasive testing.	True	94 (100%)
NIPT has a better detection rate for Trisomy 21 than FTS.	True	85 (90.4%)
NIPT has a lower false-positive rate for Trisomy 21 than FTS.	True	69 (73.4%)
In which of the following scenarios is NIPT more likely to fail (i.e. give a “no call” result)?		
Foetal aneuploidy	True	65 (69.1%)
Hypertension	False	90 (95.7%)
Low maternal body mass index (BMI)	False	81 (86.2%)
Type 2 Diabetes Mellitus (DM)	False	58 (61.7%)
Systemic lupus erythematosus (SLE)	True	39 (41.5%)

## Discussion

This is the first nationwide survey of obstetrician attitudes, knowledge and practice with regard to first trimester screening and NIPT in Singapore. The Singapore Ministry of Health recently issued guidelines classifying NIPT as “Level 2 Genetic Tests” that require the skills of “appropriately trained medical practitioners” to be correctly ordered and interpreted, and to correctly explain the results and implement follow up plans [19]. Our study thus provides a timely review of current clinical practice. This will aid in the planning of formal educational programs to ensure obstetricians receive appropriate training, as well as the formulation of national guidelines to serve as benchmarks for clinical practice.

Traditional first trimester screening with NT and serum biochemical markers continues to be the main first-line Down syndrome screening method offered to general obstetric patients with singleton and multiple pregnancies. However almost half the respondents also offered NIPT as an alternative first-line screening option for singleton pregnancies. While Trisomy 21, 18 and 13 comprise a smaller proportion of chromosomal abnormalities in the general obstetric population and the PPVs are lower [20], there are merits to this approach as the PPVs are still superior to those of FTS [2]. Previous studies have also found that low-risk women value NIPT for providing psychological reassurance [21]. Recently updated guidelines from the American College of Obstetricians and Gynaecologists (ACOG) and the American College of Medical Genetics and Genomics (ACMG) endorse NIPT as a first-line screen for the general obstetric population as well [22, 23]. Obstetricians offering NIPT as a first-line screening option should advise patients to also have a first-trimester ultrasound and NT measurement so that structural abnormalities are not missed [24]. The NT measurement should however not be used to calculate separate risk estimates and chromosomal microarray should be considered if NT is greater than 3.5 mm [20].

A significant proportion of providers offered FTS together with NIPT (35.1%) to their patients with singleton foetuses. Simultaneous testing with multiple screening methodologies is not recommended due to poor cost-effectiveness [22]. Obstetricians may however decide to offer this approach to patients as a means of obtaining a “backup” result in case of NIPT failure, or to give them greater psychological reassurance. Patients should be counselled that concurrent FTS and NIPT increases the overall false positive rate, which may lead to unnecessary anxiety and invasive testing with the risk of miscarriage. Patients who desire diagnostic certainty and maximum detection of foetal genetic diagnoses should be counselled that normal screening results do not guarantee a “healthy” baby, and that invasive testing with chromosomal microarray (CMA) in the first instance may be a more appropriate option [23].

Contingent use of NIPT for patients with a positive FTS is cost-effective but there is currently no consensus on the most suitable FTS cut-off [20, 24]. Responses to our survey questions on options offered at varying FTS risk levels reflected a similar diversity of practice. Drawbacks of the contingent model include a possible delay in reaching a definitive diagnosis, and a 2% residual risk of a chromosomal abnormality when a normal NIPT follows an abnormal FTS result [22, 25]. Expert opinion also recommends against NIPT when patients have a very high risk FTS result (> 1:10) as Trisomy 21, 18 and 13 contribute to only 70% of chromosomal abnormalities in this population [24]. Further research is required to refine guidelines for the incorporation of NIPT into a contingent screening model; the United Kingdom National Screening Committee (UKNSC) is evaluating the role of NIPT when the FTS risk for Trisomy 21 is greater than 1 in 150 [26], and its findings may provide better direction.

A sizeable proportion of obstetricians offered NIPT as first-line screening for twin pregnancies. A recent meta-analysis provides good evidence to suggest that the performance of NIPT for trisomy 21 in twin pregnancies may be similar to that for singletons [27]. Single nucleotide polymorphism (SNP)-based NIPT methods may be used for twin pregnancies [22]. The improved detection rate and lower false positive rate of NIPT for trisomy 21 may help to reduce the need for invasive testing, which has a higher miscarriage risk in twin pregnancies. Patients should however understand that the NIPT failure rate is higher for twins [28] as the foetal fraction from each twin is lower.

The clinical knowledge section was designed to evaluate fundamental knowledge expected of practitioners offering NIPT to screen for the common trisomies, and covered domains such as performance characteristics (questions 16a-c, e, f) and practical applications (questions 16d and q17). It was therefore encouraging that the median score was relatively high at 10 out of 12. Overall knowledge scores did not differ significantly between MFM and non-MFM specialists, nor between specialists and residents. This differs from previous studies that show MFM specialists to be significantly more knowledgeable than non-MFM specialists, and senior fellows to have superior knowledge scores compared to junior fellows [15]. This is a positive finding as both MFM and non-MFM obstetricians at all levels of training see and counsel pregnant women on the options for first trimester screening in Singapore.

We consider knowledge that NIPT is a screening test requiring confirmation with karyotype [23, 24] to be essential for offering this test so as to prevent the disaster of wrongful pregnancy termination. It was thus reassuring that all respondents correctly answered that high risk NIPT results need to be confirmed with invasive testing (100%), and that the majority indicated that NIPT is not a diagnostic test for Trisomy 21 (96.8%).

Only 69.1 and 41.5% respectively were aware that NIPT was more likely to be fail in foetal aneuploidy [3, 22, 29] and in patients with SLE [8, 30]. The former scenario is important as a no-call result due to low foetal fraction in the maternal circulation is associated with an increased risk of foetal aneuploidies such as Trisomy 13, 18, monosomy X and triploidy [28, 31]. Trisomy 21 was also seen in 23% of cases of low foetal fraction in one prospective study [3], though other reports have suggested that the foetal fraction in pregnancies affected by Down syndrome is in fact unchanged or higher when compared to pregnancies with euploid foetuses [32, 33]. Women should be carefully counselled and diagnostic testing considered if a no-call NIPT test was done at an appropriate gestational age [23].

Patients with SLE have a higher chance of test failure as maternal cfDNA levels are increased by inflammatory conditions. This results in a corresponding drop in foetal fraction. We included SLE in our test of knowledge as it is more prevalent among females and Asians [34]. These patients should be informed that they may miss the window for first trimester screening if they opt for NIPT but fail to receive a result.

56.4% (53/94) of respondents reported offering their patients screening for SCAs because they wanted to give patients the autonomy of choice and 55.3% (52/94) because there was no additional cost for offering this option. As the detection rate of NIPT for SCAs is > 90%, false-positive rate ~ 1% and PPV ~ 48.4%, which is higher than that for Trisomy 21, 18, 13 with conventional first trimester screening [23], informing women that they have the option to use NIPT to screen for SCAs appears to be reasonable practice. Practically, however, patients are likely to be poorly informed about SCAs, these conditions can have variable presentations and prognosis, and there is a potential for discovery of maternal or foetal sex chromosomal abnormalities of uncertain clinical significance [28]. Greater time and effort needs to be spent on counselling to enable patients to make an informed choice.

Screening for microdeletions and microduplications is more controversial. Proponents acknowledge that some of these conditions can be severe and affected children can benefit from early diagnosis and intervention. Opponents argue that offering screening for these conditions is unethical, as there are insufficient clinical utility studies to provide proper pre-test counselling and the variable expressivity and penetrance of the conditions screened for make it challenging to counsel patients with high-risk results [12, 23]. Screening for a greater number of CNVs also increases the risk of false positive and negative results, which can lead to increased harm in the form of patient uncertainty and anxiety, and more invasive testing procedures. A more nuanced approach can perhaps be considered, with avoidance of genome-wide CNV screening in favour of targeted NIPT for selected CNVs (e.g. 22q microdeletion) which have more clinical validation data, and also explaining the alternative of diagnostic testing with karyotype and CMA for patients who wish to test for a wider range of foetal genomic conditions [23].

Two NIPT platforms dominated amongst respondents, largely because they were available in the centres of practice. Harmony was also one of the first NIPT platforms introduced locally. The public hospitals in Singapore, where two-thirds of our respondents practiced, generally offer only one or two NIPT platforms with good clinical validity. This simplifies the selection process for obstetricians, and a familiar reporting format aids in interpretation of results. Market dominance may however result in inflated costs and indirectly influence clinical practice. For instance, clinicians may offer expanded screening simply because the NIPT platform with the largest market share lists it as a screening option; indeed, over half of our respondents reported offering their patients screening for SCAs and CNVs because “there was no extra cost”. One way to guard against this is to have a review committee that periodically evaluates the available NIPT platforms and selects the most clinically appropriate and cost-effective one(s) for use in the centre.

### Limitations

Our survey response rate was 30.7%. This compares favourably to previously published surveys of healthcare professionals, which have response rates varying from 15.9 to 36.5% [9, 15–17], but may limit the generalizability of the results. Non-specialists and consultants practising in public hospitals were also over-represented. Nonetheless, as our respondents encompassed a good cross-section of practicing obstetricians, our results give us information about knowledge, attitudes and practice across different levels of expertise, practice settings and percentages of clinical load in obstetrics. The knowledge section focused on fundamental aspects of NIPT that the authors felt were crucial prerequisites for offering the test. More in-depth exploration of practice behaviours found in our study, such as reasons for offering certain recommendations (e.g. concurrent FTS and NIPT), the limitations of NIPT in screening for SCAs and CNVs and information on counselling time and personnel, would be informative and may be achieved by using qualitative research methods in a follow-up study.

## Conclusions

Our findings show the diversity of clinical practice with regard to the incorporation of NIPT into prenatal screening algorithms, and suggest that the use of NIPT both as a first-line screening tool in the general obstetric population, and to screen for SCAs and CNVs, is becoming increasingly prevalent. Clear guidance and continuing educational support are essential for ensuring that providers in this rapidly evolving field are appropriately trained. Issues that should be covered include the benefits and disadvantages of various screening options, next-steps after abnormal FTS results, performance characteristics of NIPT for SCAs and CNVs, and understanding populations for whom NIPT would be more likely to fail.

The complexity of counselling will only increase as NIPT technology improves and the range of conditions that can be screened for expands. Formal educational programs in prenatal screening and counselling should be made compulsory and national guidelines formulated to serve as a benchmark for clinical practice. Access to foetal medicine specialists and genetic counsellors should be improved for cases where more complex counselling is required. Tighter regulation of laboratories, with mandatory reporting of detection rates and PPVs, will aid in the informed consent process [5] and should be considered at a national level.

## Supplementary information

**Additional file 1.** Questionnaire

**Additional file 2.** CHERRIES Checklist

## Data Availability

The datasets used during the current study are available from the corresponding author on reasonable request.

## Abbreviations

NIPT: Non-invasive prenatal testing
cfDNA: Cell-free DNA
PPV: Positive predictive value
FTS: First trimester screening
NPV: Negative predictive value
NT: Nuchal translucency
SCAs: Sex chromosomal aneuploidies
CNVs: Microdeletion/duplication syndromes
MFM: Maternal-foetal medicine
MCDA twins: Monochorionic diamniotic
DCDA twins: Dichorionic diamniotic
BMI: Body mass index
DM: Diabetes Mellitus
SLE: Systemic lupus erythematosus
ACOG: American College of Obstetricians and Gynaecologists
ACMG: American College of Medical Genetics and Genomics
CMA: Chromosomal microarray
SNP: single nucleotide polymorphism
