# Supplementary material for: Prenatal screening in the era of non-invasive prenatal testing: a Nationwide cross-sectional survey of obstetrician knowledge, attitudes and clinical practice
Source: BMC Pregnancy Childbirth. 2020 Oct 1;20:579. doi: 10.1186/s12884-020-03279-y (PMC7528474; doi:10.1186/s12884-020-03279-y)
Supplement: Supplementary file 1 — Additional file 1. Questionnaire [file 12884_2020_3279_MOESM1_ESM.docx]

**Additional File 1: Questionnaire**

**Prenatal Screening in the Era of NIPT: A Survey of Obstetricians**

Thank you for participating in our survey. It will take approximately 15 minutes to complete.

STUDY IN BRIEF

We would like to learn what obstetricians know about NIPT, and find out more about your attitudes and clinical practice with regard to Down syndrome screening. The information derived from this study will additionally help the College of O&G to better tailor our series of Prenatal Genetics lectures.

CONSENT & CONFIDENTIALITY

This project has been approved by the Singhealth IRB. By completing and returning this questionnaire, you consent to participate in our study. All questionnaires are anonymous and no identifying information will be collected.

INSTRUCTIONS

Please either fill in the attached PDF file or click here for an online link. Note that some questions invite you to check ALL answers that apply.

You are welcome to contact Dr Yang Liying at [yang.liying@singhealth.com.sg](mailto:yang.liying@singhealth.com.sg) if you have any questions regarding this survey.

**SECTION 1: DEMOGRAPHICS**

1. What is the practice setting in which you see the majority of your patients? Choose an item.
2. What is your level of expertise? Choose an item.
3. How many years have you been in practice? Choose an item.
4. Approximately what percentage of your practice is in obstetrics? Choose an item.
5. Are you a specialist in maternal-fetal medicine? Choose an item.

**SECTION 2: CLINICAL PRACTICE**

1. How comfortable are you in discussing the following with your patients? **Please select an option from 1 to 5, with 1 being Not comfortable and 5 being Very comfortable.**

|  |  |  |
| --- | --- | --- |
|  | Clinical features of Trisomy 21 | Choose an item. |
|  | Accuracy and limitations of NIPT for T21, T18 and T13 | Choose an item. |
|  | Accuracy and limitations of NIPT for sex chromosome aneuploidies | Choose an item. |
|  | Options if the patient receives a high risk NIPT result | Choose an item. |

1. What is your primary objective when offering prenatal screening for your general obstetric patients?

|  | Screening for Trisomy 21 |
| --- | --- |
|  | Screening for the common fetal aneuploidies (T21, T18, T13) |
|  | Screening for as many fetal conditions as possible (e.g. sex chromosome aneuploidies, microdeletions, microduplications) |
|  | Others  Click here to enter text. |

1. Which of the following do you offer to your general obstetric patients as first-line screening methods for Down syndrome? **Please check all that apply.**

|  |  | Singleton pregnancy | DCDA Twins | MCDA Twins |
| --- | --- | --- | --- | --- |
| a) | Nuchal translucency (NT) alone |  |  |  |
| b) | First trimester screening (NT, PAPPA, bHCG) alone |  |  |  |
| c) | NIPT |  |  |  |
| d) | NT in combination with NIPT |  |  |  |
| e) | FTS in combination with NIPT |  |  |  |
| f) | Invasive testing |  |  |  |
| g) | Others  Click here to enter text. |  |  |  |

1. Which NIPT platforms do you offer to your patients? **Please check all that apply.**

|  | Harmony |
| --- | --- |
|  | iGene |
|  | Panorama |
|  | Others  Click here to enter text. |

1. Why did you choose these platforms? **Please check all that apply.**

|  | It is the default platform offered by my institution. |
| --- | --- |
|  | It has superior test performance. |
|  | It allows me to screen for more genetic conditions. |
|  | It has a lower cost. |
|  | There is comprehensive laboratory support (e.g. queries on no-call results) |
|  | Other Click here to enter text. |

1. For patients who opt for NIPT, do you recommend screening for sex chromosome aneuploidies? **Please check all that apply.**

|  | Yes, as I want to give my patients the autonomy to screen for more genetic conditions. |
| --- | --- |
|  | Yes, as there is no extra cost. |
|  | No, because there is low positive predictive value. |
|  | No, because there is a low detection rate. |
|  | Others  Click here to enter text. |

1. For patients who opt for NIPT, do you recommend screening for microdeletions and microduplications (e.g. 22q deletion)? **Please check all that apply.**

|  | Yes, as I want to give my patients the autonomy to screen for more genetic conditions. |
| --- | --- |
|  | Yes, as there is no extra cost. |
|  | No, because there is low positive predictive value. |
|  | No, because there is a low detection rate. |
|  | Others  Click here to enter text. |

1. What options would you offer in each of the following scenarios? **Please check all that apply.**

|  |  | No further testing | NIPT | Invasive testing | Termination of pregnancy | Other |
| --- | --- | --- | --- | --- | --- | --- |
|  | The adjusted risk for T21 is 1:670 on FTS. |  |  |  |  | Click here to enter text. |
|  | The adjusted risk for T21 is 1:86 on FTS. |  |  |  |  | Click here to enter text. |
|  | The adjusted risk for T21 is 1:7 on FTS. |  |  |  |  | Click here to enter text. |

1. What do you recommend if your patient has a high risk NIPT result for Trisomy 21?

|  | Repeat NIPT |
| --- | --- |
|  | Invasive testing |
|  | Termination of pregnancy |
|  | Others  Click here to enter text. |

**SECTION 3: CLINICAL KNOWLEDGE**

1. NIPT detects cell-free DNA in the maternal bloodstream. Where does this cell-free DNA originate from?

|  | Fetal erythroblasts |
| --- | --- |
|  | Fetal skin cells |
|  | Placental cells |

1. Are the following statements true or false?

|  |  | **True** | **False** | **Unsure** |
| --- | --- | --- | --- | --- |
|  | NIPT can be offered from 8 weeks of pregnancy. |  |  |  |
|  | All the chromosomal abnormalities diagnosed via amniocentesis can be detected via NIPT. |  |  |  |
|  | NIPT is a diagnostic test for Trisomy 21. |  |  |  |
|  | A high risk NIPT result should be confirmed with invasive testing. |  |  |  |
|  | NIPT has a better detection rate for Trisomy 21 than FTS. |  |  |  |
|  | NIPT has a lower false-positive rate for Trisomy 21 than FTS. |  |  |  |

1. In which of the following scenarios is NIPT more likely to fail (i.e. give a “no call” result)? **Please check all that apply.**

|  | Fetal aneuploidy |
| --- | --- |
|  | Hypertension |
|  | Low maternal BMI |
|  | Type 2 diabetes mellitus |
|  | Systemic lupus erythematosus |

**Thank you for completing the survey.**

Please enter any additional thoughts or comments that you have in the box below.

| Click here to enter text. |
| --- |
