# Supplementary material for: Prenatal screening in the era of non-invasive prenatal testing: a Nationwide cross-sectional survey of obstetrician knowledge, attitudes and clinical practice
Source: BMC Pregnancy Childbirth. 2020 Oct 1;20:579. doi: 10.1186/s12884-020-03279-y (PMC7528474; doi:10.1186/s12884-020-03279-y)
Supplement: Supplementary file 2 — Additional file 2. CHERRIES Checklist [file 12884_2020_3279_MOESM2_ESM.docx]

**Additional File 2: CHERRIES Checklist for Online Questionnaires**

| Study Design: Describe target population & sample frame. Is the sample a convenience sample? | It is not a convenience sample.  All obstetricians in the Singapore specialist register will be invited to participate. |
| --- | --- |
| IRB Approval: Has the study been approved by an IRB? | Yes, approved by the Singhealth IRB. |
| Informed consent:  Describe the informed consent process. Where were the participants told the length of time of the survey, which data were stored & where/for how long, who the investigator was, purpose of the study? | The introductory paragraph at the start of the survey will   - Describe the purpose of our study - Inform participants that no identifying data will be collected - Provide the PI’s contact info   They will also be informed that participating in the survey will imply their consent for their answers to be used for research. |
| Data protection: If any personal information was collected or stored, describe what mechanisms were used to protect unauthorized access. | No personal information will be collected or stored. |
| Development and testing | The survey was reviewed for content validity by 3 consultants in Maternal Fetal medicine. It was pilot tested by a group of 15 obstetrician residents and consultants. Questions were edited for clarity after pilot testing. |
| Open survey versus closed survey | This is a closed survey.  Participants will have to enter a password to access the survey. |
| Contact mode:  Indicate whether or not the initial contact with the potential participants was made on the Internet. | Contact will be made via email. |
| Advertising the survey:  How/where was the survey announced or advertised? It is important to know the wording of the announcement as it will heavily influence who chooses to participate. | The survey will be advertised via email sent by the College of O&G.  *“We would like to invite you to participate in a survey on Prenatal Screening in the era of NIPT. The aim of our survey is to learn what obstetricians know about NIPT, and find out more about your attitudes and clinical practice with regard to Down syndrome screening. The information derived from this study will additionally help the College of O&G to better tailor our series of Prenatal Genetics lectures.”* |
| Web/Email:  State the type of e-survey (e.g. one posted on a Web site or one sent out through e-mail). If it is an e-mail survey, were the responses entered manually into a database, or was there an automatic method for capturing responses? | The survey will be sent out in two forms, an e-survey on Google Forms and a Word document. Participants can choose either mode of completion.  We decided to use two formats to increase the response rate, as participants will be able to choose the format they are more comfortable with. |
| Context:  Describe the Web site (for mailing list/newsgroup) in which the survey was posted. | N/A. Participants will access the site via the link sent to their email. |
| Mandatory/voluntary:  Was it a mandatory survey to be filled in by every visitor who wanted to enter the Web site, or was it a voluntary survey? | N/A. |
| Incentives:  Were any incentives offered (e.g., monetary, prizes, or non-monetary incentives such as an offer to provide the survey results)? | No incentives will be offered. |
| Time/Date:  In what timeframe were the data collected? | The data will be collected over a period of 1 month. |
| Randomisation of items:  To prevent biases items can be randomized or alternated. | The items will not be randomized as this may confuse the participants. |
| Adaptive questioning:  Use adaptive questioning (certain items, or only conditionally displayed based on responses to other items) to reduce number and complexity of the questions. | N/A. |
| Number of Items:  What was the number of questionnaire items per page? The number of items is an important factor for the completion rate. | Google Form  Number of questions per section  5  12 (section 2-1)  4 (section 2-2)  8  Microsoft Word Form  5  9  3 |
| Number of Screens:  Over how many pages was the questionnaire distributed? The number of items is an important factor for the completion rate. | Google Form  Total of 6 pages  Introduction  4 pages for survey questions  Comments page  Microsoft Word Form  Total of 7 pages  Introduction  6 pages for survey questions and comments |
| Completeness check:  Were consistency or completeness checks before questionnaire submission? An alternative is to check for completeness after the questionnaire has been submitted (and highlight mandatory items). | Yes there was built in completeness check. |
| Unique site visitor | We will only calculate participation rate.  The following formula will be used: Number of completed surveys / number of invited participants |
| View rate (Ratio of unique survey visitors/unique site visitors) | N/A |
| Participation rate | N/A |
| Completion rate (Ratio of users who finished the survey/users who agreed to participate): This is only relevant if there is a separate “informed consent” page or if the survey goes over several pages. This is a measure for attrition. | This will not be calculated. |
| Cookies used:  Indicate whether cookies were used to assign a unique user identifier to each client computer. | We did not incorporate these elements into our survey. |
| IP check:  Indicate whether the IP address of the client computer was used to identify potential duplicate entries from the same user. |  |
| Log file analysis:  Indicate whether other techniques to analyze the log file for identification of multiple entries were used. If so, please describe. | We decided not to require log-in as this may reduce participation rate due to perceived non-anonymity of responses. |
| Registration:  In “closed” (non-open) surveys, users need to login first and it is easier to prevent duplicate entries from the same user. |  |
| Handling of incomplete questionnaires:  Were only completed questionnaires analyzed? | All submitted questionnaires will be analysed.  If certain questions were not completed by all participants, this will be reported in our results. |
| Statistical correction:  Indicate whether any methods such as weighting of items or propensity scores have been used to adjust for the non-representative sample; if so, please describe the methods. | No statistical correction will be used to adjust for the non-representative sample. |
